# Analysis of Cobalamin (Vit B12) in Ripened Cheese by Ultra-High-Performance Liquid Chromatography Coupled with Mass Spectrometry

**DOI:** 10.3390/foods11182745

**Published:** 2022-09-07

**Authors:** Giulia Rampazzo, Elisa Zironi, Giampiero Pagliuca, Teresa Gazzotti

**Affiliations:** 1 Department of Veterinary Medical Science, Alma Mater Studiorum University of Bologna, Via Tolara di Sopra 50, 40064 Ozzano dell’Emilia, Italy; 2 Health Sciences and Technologies-Interdepartmental Centre for Industrial Research (CIRI-SDV), University of Bologna, 40064 Ozzano dell’Emilia, Italy

**Keywords:** cobalamin, vitamin B12, ripened cheese, UHPLC-MS/MS

## Abstract

The analysis of natural cobalamins in dairy products still represents an analytical challenge. The matrix’s complexity, low concentration level, light sensitivity, and binding to proteins are just some of the aspects that make their quantification a difficult goal to achieve. Vitamin B12 plays a fundamental role in human nutrition, and its intake is closely linked to a diet that includes the consumption of food of animal origin. In the current literature, few studies have been carried out on the quantitation of cobalamin in ripened cheeses. A sensitive, selective, and robust ultra-high-performance liquid chromatography coupled with tandem mass spectrometry (UHPLC-MS/MS) method was developed, validated, and applied on ripened cheeses from different species (cow, sheep, and goat) purchased from local Italian markets, highlighting species-dependent differences in vitamin B12 concentrations. The vitamin B12 extraction procedure was performed by converting all cobalamins to the cyanocobalamin form. Furthermore, solid-phase extraction was used for matrix clean-up and analyte preconcentration. The proposed method showed good performance in terms of linearity, sensitivity, reproducibility, and repeatability. The mean vitamin B12 content ranged from <LOQ to 38.9 ng/g. Sheep cheese showed the highest concentrations of vitamin B12, with a mean content of 29.0 ng/g.

## 1. Introduction

Cobalamins are a group of water-soluble compounds known collectively as vitamin B12 (vit B12). Vitamin B12 has a complex structure containing cobalt as the central ion in a four-membered planar pyrrolic ring (corring ring). The main natural forms of cobalamin found in food are hydroxocobalamin, 5c-deoxyadenosylcobalamin, methylcobalamin, and cyanocobalamin, which are present only in small amounts [1]. Cyanocobalamin is more stable because it is less light-sensitive than the other forms. Vitamin B12 plays an important role in human nutrition and is critical for optimal blood cell construction, DNA synthesis, and maintaining healthy myelin sheets of nerve cells. It is, therefore, a key micronutrient necessary for proper brain development [2,3]. According to the European Food Safety Authority [4], the recommended adequate intake of cobalamin is 4 μg/day with a range of 4.2 to 8.6 μg/day in adults in several EU countries, with a higher requirement during pregnancy and lactation. Vitamin B12 intake is strictly linked to a diet that includes the consumption of animal-derived foods, such as dairy products, meat, fish, and eggs. Furthermore, Vogiatzoglou et al. (2009) [5] reported that vitamin B12 in dairy products plays a key role in human nutrition due to its greater bioavailability than other foods of animal origin. Plasma vitamin B12 intake has been associated with increasing vitamin B12 intake from dairy or fish consumption but not with vitamin B12 intake from meat or eggs. For the same vitamin B12 content, intake from dairy products led to the greatest increase in plasma vitamin B12. Cobalamin deficiency is common worldwide and can lead to anemia and neurological dysfunctions in humans. Certain population groups, such as infants and older adults, may be prone to vitamin B12 deficiency. A higher risk is expected, especially for people who eat diets rich in plant-based food and low in animal-based foods [6].

In ruminants, vitamin B12 is synthetized in the presence of cobalt by rumen microflora (bacteria and archaebacteria), then adsorbed and stored in their bodies, making milk and other tissues particularly rich in B12 [7]. According to the National Food Composition databases of Europe, Switzerland, and the USA [8,9,10], cobalamin concentration in milk and dairy products varies between species, with sheep’s and cow’s milk proving to be the richest with concentrations ranging from 4.4 to 7.0 ng/g and 4.5 to 5.4 ng/g, respectively. The concentration of vit B12 in goat milk was found to be the lowest, with values ranging from 0.4 to 0.9 ng/g. Consequently, the cyanocobalamin content in cheese varies, ranging from 1 to 30 ng/g, as reported in a few works [11,12,13]. Thus, the species of milk source and the characteristics of the cheese-making process can influence the final amount of vit B12 in cheese.

Vitamin B12 analysis can be based on two approaches: microbiological and chemical. The microbiological tests generally involve the use of *Lactobacillus delbrueckii* [14]; these methods were originally developed for the analysis of vitamin preparations but are also applied to fortified foods and food matrices in general. Regarding chemical–physical methods, spectrophotometry is applicable, but with a complex matrix, it is difficult to achieve good sensitivity [15]. According to the literature, most of the studies on the chemical quantification of vitamin B12 in different matrices are based on radioisotopic assays [16] or chromatographic techniques combined with UV [17,18,19,20], fluorescence [21], or diode array detectors [22]. Radioisotopic methods are accurate and sensitive but expensive because of the need to use radio-labeled cyanocobalamin. Another method is electroluminescent (ECL), which involves the use of highly reactive materials. Inductive-coupled plasma-mass spectrometry (ICP-MS) is also routinely used in various research [15,23]. ICP-AES (inductively coupled plasma atomic emission spectroscopy) was also used by Bartosiak et al. (2018) [24] for the determination of cobalt species in dietary supplements. In recent times, several LC-electrospray ionization (ESI)-mass spectrometry methods have been developed for the determination of vitamin B12 concentrations in food products [14,25,26,27]; these techniques are more sensitive and selective, allowing researchers to quantify naturally occurring cobalamin in food at very low levels. However, most of the LC-electrospray ionization-mass spectrometry methods available in the literature for the determination of cobalamin have been developed on matrices with a medium–high water content or plant-based matrix. A limited—and not recent—number of works are currently available on methods developed in complex matrices, such as ripened cheese. In recent works, the extraction of cobalamins in food used potassium cyanide (KCN) boiling step to convert all cobalamins to the stable form, cyanocobalamin [18,19,25,28,29].

In general, cobalamin is present in foods in very low concentrations, and in milk and dairy products, it is mostly protein-bound. Furthermore, treatments undergone by milk during processing cause various losses of vitamin B12. Removal of the whey fraction, generally carried out during the cheese-making process, causes severe loss (30–80%) of vitamin B12 due to its solubility in water [13]. Arkbage et al. (2003) [16] also tested the effect of heat treatment and boiling on vit B12 concentration in milk. Temperatures of 76 °C for 16 s and 96 °C for 5 min did not cause B12 losses. However, high losses were observed in other studies [30,31]: boiling milk for 2–5 min and 30 min resulted in vitamin B12 reductions of 30% and 50%, respectively, as were minor losses (less than 10%) of vitamin B12 after pasteurization and 0–20% loss after ultra-high-temperature treatment. Packaging and storage can also alter vitamin B12 concentration; ultra-high-temperature-treated milk stored at 7 °C for 18 weeks did not show losses of vit B12, while storage at 23 °C and 35 °C for 18 weeks caused a significant reduction of up to 33% of the vitamin B12 concentration. Vitamin retention is also highly dependent on the oxygen concentration in the package [13]. Because of their natural presence in the matrix, their low concentration level, their binding to proteins, and the difficulties arising from matrix characteristics, cobalamin quantification in ripened cheese presents an analytical challenge. Considering the importance of cobalamin in human nutrition (also in light of current changes in lifestyle and dietary habits), its high bioavailability in dairy products, and the lack of available specific methods on cobalamin quantitation in ripened cheese and the resulting lack of data, the aim of the present work was to: (1) develop and (2) validate a method for the determination and quantitation of vitamin B12 in ripened cheese samples, then (3) apply the proposed method to real cheese samples from different species using ultra-high-performance liquid chromatography coupled with tandem mass spectrometry (UHPLC-MS/MS). The preliminary data obtained contribute to highlighting species-dependent differences in vitamin B12 concentration.

## 2. Materials and Methods

### 2.1. Reagents and Chemicals

Cyanocobalamin (purity ≥ 99.0%) and methotrexate (internal standard, IS) (purity ≥ 98.0%) were purchased from Sigma-Aldrich (St. Louis, MO, USA). Acetonitrile, methanol, and formic acid, all LC-MS grade, were acquired by Merck (Darmstadt, Germany), and ammonium formate and sodium acetate were purchased from Sigma-Aldrich (St. Louis, MO, USA). Analytical-grade potassium cyanide (Carlo Erba Reagenti SpA, Rodano, Italy) and glacial acetic acid (Merck KGaA, Darmstadt, Germany) were used. n-hexane was purchased from Merck KGaA, Darmstadt, Germany. Ultrapure water was freshly produced from a Milli-Q^®^ water purification system (Merck, Darmstadt, Germany). Oasis HLB 200 mg/6 cc cartridges acquired from Waters Corp. (Milford, MA, USA) were used for solid-phase extraction (SPE). PTFE syringe filters (13 mm 0.2 µm) were purchased from Waters Corp. (Milford, MA, USA). Stock solutions of cyanocobalamin (10 mg of standard in 100 mL of distilled water) and methotrexate (5 mg of standard in 250 mL of distilled water) were prepared. Working solutions were prepared by diluting stock solutions appropriately in water to reach a concentration of 1 µg/mL. All the solutions were stored and refrigerated at 4 °C.

### 2.2. Sampling

Different types of ripened cheeses (*n* = 12) were purchased from local Italian markets. Five (*n* = 5) cheeses were made from cow milk, four (*n* = 4) from sheep milk, three (*n* = 3) from goat milk. All the collected cheeses were divided into aliquots, grated, and stored at −20 °C until their analysis.

### 2.3. Sample Preparation

The extraction procedure was performed in accordance with the protocol described by Zironi et al. (2013) [29], with some modifications due to the high lipid component characterizing the matrix. All procedures were carried out in dim light to avoid vit B12 degradation due to its photosensitivity. Firstly, 1 g of ground cheese was weighed in 15 mL polypropylene tube and fortified with 25 μL of internal standard solution at 1 μg/mL; 3 mL of distilled water was added, and the sample was then homogenized with ultraturrax for 2 min. The content was transferred in a 10 mL glass tube. Thus, 3 mL of sodium acetate buffer (200 mM, adjusted to pH 4 with glacial acetic acid) and 75 μL of 1% potassium cyanide solution in water were added. The sample was then left in a water bath at 90 °C for an hour under constant magnetic agitation. This step is essential for the conversion of all forms of cobalamin to cyanocobalamin and the release of all protein-bound cobalamins. Since potassium cyanide reacts with acids to form highly toxic and rapid-acting hydrogen cyanide gas, all operations must be carried out under a fume cupboard while wearing nitrile gloves, a lab coat, and safety glasses. Residues were destroyed with alkaline NaOCl solution. Sample was transferred in a 15 mL polypropylene tube, and 5 mL of hexane was added, followed by a centrifugation step at 20 °C 9000 rpm for 10 min. Thus, the supernatant was eliminated, another 5 mL of n-hexane was added, and the centrifugation step was repeated. After the elimination of the supernatant, the clean-up was performed by solid-phase extraction (SPE) using Oasis HLB cartridge. The conditioning of the cartridge was carried out with acetonitrile and water. Next, the sample was loaded, washed with 2 mL of water, 2 mL of water: methanol (95:5), and then eluted with 2 mL of an acetonitrile:water (50:50) solution. The eluted was finally filtered with PTFE syringe filter (13 mm, 0.2 µm) and diluted with water (1:5) prior to the UHPLC-MS/MS injection.

### 2.4. UHPLC-MS/MS Analysis

The quantification of cyanocobalamin in ripened cheese was performed using ultra-high-performance liquid chromatography coupled with triple–quadrupole mass spectrometry (UHPLC-MS/MS) technology. The equipment employed consisted of a Waters Acquity UHPLC^®^ binary pump coupled with a Waters Xevo^®^ TQ-S micro triple–quadrupole mass spectrometer (Waters Corporation, Milford, MA, USA) equipped with an electrospray ionization source (ESI). Analyses were performed in positive electrospray ionization (ESI+) mode and MRM (multiple reaction monitoring) modes, following two specific transitions for the target analytes. MS parameters of the compounds are summarized in Table 1. The transition 678.43 > 147.15 of cyanocobalamin was used for the quantitation. ESI capillary voltage was set at +0.90 kV, source temperature at 150 °C, and desolvation temperature at 600 °C. Desolvation and cone gas flow were 800 and 80 L/h, respectively, and argon was used as collision gas. The chromatographic conditions were settled as follows: mobile phases were 5 mM ammonium formate in water acidified with 0.05% of formic acid (A) and acetonitrile with 0.3% of formic acid (B). The gradient started from 0 min with 100% phase A; this percentage decreased linearly to 95% in 2 min and 50% in 3 min until it reached 0% in 1 min. This condition was held for 1 min, then increased to 100% in 0.50 min and held for 2.5 min for column re-equilibration. Total run time was 10 min, the flow rate was 0.350 mL/min, and the volume injected was 10 μL. The chromatographic separation was carried out on a Waters Acquity BEH C_18_ UHPLC^®^ (50 × 2.1 mm, 1.7 μm) column (Waters Corporation, Milford, MA, USA) maintained at 40 °C. The thermostated autosampler was kept at 20 °C. Data were acquired and processed using Waters MassLynx™ 4.1 software (Waters Corporation, Milford, MA, USA).

### 2.5. Method Validation

**Figure 1:**
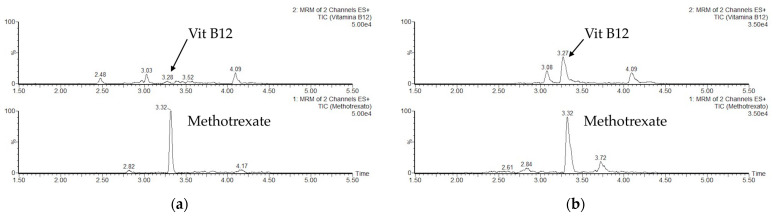
Chromatograms of: (**a**) Sample of cheese with the lowest natural B12 content spiked with 25 ng/g of methotrexate (IS); (**b**) Sample of cheese spiked with 25 ng/g of cyanocobalamin and 25 ng/g of methotrexate (IS).

The proposed LC–MS/MS method was validated in accordance with in-house criteria for specificity, linearity, trueness, precision, and limit of quantification (LOQ) considering as guidelines the Commission Implementing Regulation (EU) 2021/808 [32]. Moreover, standard addition method was used to compensate for matrix effects. Specificity was demonstrated by injecting cheese samples and fortified samples with cyanocobalamin standard and IS at a concentration of 25 ng/g; the chromatograms showed a significant increase in peak area intensity at the specific retention time of the compounds. To determine trueness and precision, 1 g of cheese sample was spiked at three different concentrations (0, 25, and 50 ng/g) with standard cyanocobalamin solution and a fixed amount of methotrexate (25 ng/g). The spiked samples were extracted in triplicate (*n* = 9) over three non-consecutive days to determine the repeatability (RSD_r_; intra-day precision) and within-lab reproducibility (RSD_R_; inter-day precision). Trueness, expressed as bias, is the relative difference between the mean measured value and the spiked concentration. The precision was measured as relative standard deviation to the mean of both repeatability and reproducibility. The calibration curve was prepared with 1 g cheese spiked with appropriate amounts of the standard cyanocobalamin working solution (1 μg/mL) to obtain 6 levels of concentration (0, 6, 12, 25, 50, and 100 ng/g); 25 μL of the internal standard methotrexate working solution (1 μg/mL) was added to each sample (Figure S1). After the analysis of all cheeses, the cheese with the lowest natural vit B12 content was chosen for the construction of a matrix-matched calibration curve used for method validation and quantification (Figure 1). The limit of quantification (LOQ) of the method was defined as the measured concentration with a signal-to-noise (S/N) ratio greater than 10.

## 3. Results and Discussion

### 3.1. Optimization of the Extraction Procedures

A methodology previously developed for milk and dairy products was chosen as a starting point. Zironi et al. (2013) [26] developed a protocol for determining cyanocobalamin concentrations in milk, mozzarella cheese, whey, and curd. Considering the difference in lipid concentration and consistency due to the low percentage of water in ripened cheeses compared to the matrices used in the previously methodology, the original extraction procedure was deeply modified. A grinding step was applied to the cheeses before their analysis. Then, due to their consistency and lipid content, water addition and a delipidation step were required. The grinding step and the water addition helped to achieve a better homogenization and extraction of samples. Adding an organic solvent for the delipidation assisted in the removal of the fat content to improve the sample clean-up and ensure higher efficiency in the SPE step. For this purpose, different volumes of water added to the ground cheese were tested—specifically 3, 4, and 5 mL. Then, different organic solvents were tested for lipid removal, especially n-hexane and diethyl ether. The use of 3 mL water and 3 mL of n-hexane gave better results in terms of homogeneity and clean-up of the samples. Furthermore, the delipidation step was tested both before and after the conversion step of cobalamins to cyanocobalamins. Moreover, the purification step by SPE was also modified from the original protocol. An additional wash with 2 mL of MeOH:H_2_O (95:5) was added. Inserting the delipidation after the conversion step and adding the extra wash with MeOH:H_2_O (95:5) gave better results in terms of analyte recovery.

### 3.2. Optimization of the Chromatographic (LC) Conditions

Chromatographic conditions were investigated to achieve optimal separation and retention of the compounds. Water was used as the polar phase, and acetonitrile (ACN) as the organic phase. Different concentrations of formic acid (FA) in both solvents were tested, in the range of 0–0.1% for water and 0–0.3% for ACN. Different amounts of ammonium formate dissolved in the polar phase were also tested. Finally, H_2_O 5 mM ammonium formate, 0.05% AF, and ACN 0.3% AF enabled the best chromatographic behavior of the analytes. Furthermore, different UHPLC columns (HSS T_3_ and BEH C_18_) were evaluated to optimize the chromatographic separation of the compounds. The BEH C_18_ column provided the best resolution and peak shape of vit B12. BEH C_18_ contributed to better results in terms of robustness and reproducibility of the analysis.

### 3.3. Method Validation

Considering the complexity of the matrix and its natural cobalamin content, a matrix-matched calibration curve was used for quantitation. The linearity of the method was proved with six-point matrix-matched calibration curves of 0–100 ng/g. Peak area ratios between cyanocobalamin and methotrexate were plotted against their concentration ratios, and a linear regression least square model was applied, showing a good linearity (R^2^ > 0.99) for vitamin B12 within the working range. Precision results as a relative standard deviation (RSD) obtained for the daily analysis of three spiked replicates at each of three concentration levels (0, 25, and 50 ng/g) ranged from −11.8% to 19.3%. The inter-day precision at the three different levels studied over three separate days (a total of *n* = 9 replicates at each concentration level) was calculated to be between −3.8% and 16.2%. The results, summarized in Table 2, showed a good performance of the method since RSD_r_ and RSD_R_ were <20%, and trueness was included in the range of −20% and +10%. Since vitamin B12 is a natural compound in cheeses, it is impossible to determine a limit of quantification (LOQ) in this matrix. The instrumental LOQ, calculated as the concentration providing a chromatographic signal with a signal-to-noise (S/N) ratio equal to 10, was 2 ng/g.

### 3.4. Application of the Method to Real Samples

The proposed method was successfully applied to analyze cyanocobalamin on twelve real ripened cheese samples of different species (cow, sheep, and goat) collected from Italian markets (Table S1). All cheeses were extracted and analyzed in triplicate. Vitamin B12 content was expressed as the mean of the three analyses ± standard deviation. The results are shown in Table 3. The total mean B12 content in the cheese samples ranged from <LOQ to 38.9 ng/g. Sheep cheese showed the highest cyanocobalamin content, threefold higher than cow cheese, with a mean content of 29.0 ng/g. Two samples of ripened goat cheese showed the lowest vit B12 content (<LOQ), while one sample of extra ripened goat cheese showed a concentration of 33.5 ng/g of vit B12. The high concentration could be related to the very low water content that characterizes extra-ripened cheeses, which enables a greater analyte concentration.

Finally, cow cheese showed a mean value of 6.5 ng/g. These values align with those published in the few articles available in the literature [11,12,13] and the data reported by the National Food Composition databases of Europe, Switzerland, and the USA [8,9,10].

It should be highlighted that it is difficult to compare other types of cheeses since the literature data are mainly on cow’s milk cheeses. However, the differences in mean concentrations measured in the various cheeses may be related to the different B12 content in the milk of origin reported in the National Food Composition databases of Europe, Switzerland, and the USA [8,9,10].

## 4. Conclusions

In the present study, a sensitive LC-MS/MS method for vitamin B12 quantification in ripened cheese was developed, validated, and applied. Considering the limited availability of methods on cobalamin quantitation in this matrix, the current work allowed us to evaluate the total concentration of cyanocobalamin in ripened cheese samples and the variation in the difference in vitamin B12 content in ripened cheeses from different species (cow, sheep, and goat). Sheep cheese proved to be the richest, with vitamin B12 concentrations threefold higher than cow cheeses. Two of the three goat cheeses had the lowest content of cyanocobalamin with concentrations <LOQ. The proposed method showed good performance in terms of linearity, sensitivity, reproducibility, and repeatability. The data collected in the present work are preliminary and not statistically significant when considering the small number of samples analyzed. However, the proposed method proved useful for quantifying vitamin B12 in a cheese matrix. Considering the importance of vitamin B12 to human health, the ongoing changes in eating habits, and its common deficiency worldwide, knowing the richest food sources of this key microelement could be useful for testing possible dietary-nutritional strategies or guidelines for those most at risk of deficiency. The application of the proposed method may allow further investigation of the vitamin B12 content in ripened cheese of different origins, and this could be an advantage for the dairy industry when promoting this type of product.

## Figures and Tables

**Table 1:** MS parameters of the compounds.

Compound	RT (min)	Precursor Ion (m/z)	Product Ion 1 (m/z)	CE (eV)	Product Ion 2 (m/z)	CE (eV)
Cyanocobalamin	3.33	678.43	147.15	18	359.15	30
Methotrexate (IS)	3.37	455.25	308.17	48	134.17	28

RT, retention time; CE, collision energy.

**Table 2:** Method validation data.

Fortification Level (ng/g)	Parameter	Day 1 (*n* = 3)	Day 2 (*n* = 3)	Day 3 (*n* = 3)	Reproducibility (Inter-Day *n* = 9)
0	Precision (RSD%)	19.3	15.8	18.2	16.2
	Trueness (bias%)	+1.4	+14.5	+10.2	+8.7
25	Precision (RSD%)	19.2	13.6	3.6	15.4
	Trueness (bias%)	−11.8	+11.9	+3.1	−1.1
50	Precision (RSD%)	2.8	11.2	11.3	9.9
	Trueness (bias%)	−9.4	−4.6	+2.6	−3.8

**Table 3:** Cyanocobalamin concentration in different types of cheese.

Cheese		Vitamin B12 (ng/g)	
Type	Original Name	Single Mean ± SD	Total Mean ± SD
Cow cheese	Caciocavallo	5.9 ± 0.6	6.5 ± 4.2
	Provolone	14.2 ± 1.2	
	Piave	5.5 ± 0.8	
	Grana	4.8 ± 0.5	
	Tête de moine	2.3 ± 0.4	
Sheep cheese	Pecorino sardo 1	21.0 ± 4.3	29.0 ± 8.5
	Pecorino sardo 2	22.5 ± 5.3	
	Pecorino toscano 1	38.9 ± 2.4	
	Pecorino toscano 2	31.1 ± 6.1	
Goat cheese	Caprino 1	<LOQ *	12.5 ± 15.8
	Caprino 2	<LOQ *	
	Caprino 3 (extra ripened)	33.5 ± 2.6	

SD, standard deviation. * For the total mean calculation these data were considered at the LOQ level.

## Data Availability

The data presented in this study are available in Supplementary Material here.

## Supplementary Materials

The following supporting information can be downloaded at: https://www.mdpi.com/article/10.3390/foods11182745/s1, Figure S1: Matrix-matched calibration curve of Vitamin B12 in ripened cheese, Table S1: Concentration of vit. B12 measured in each ripened cheese sample.
