# Supplementary material for: Analysis of Cobalamin (Vit B12) in Ripened Cheese by Ultra-High-Performance Liquid Chromatography Coupled with Mass Spectrometry
Source: Foods. 2022 Sep 7;11(18):2745. doi: 10.3390/foods11182745 (PMC9497947; doi:10.3390/foods11182745)
Supplement: Supplementary file 1 [file foods-11-02745-s001.zip › foods-1865806-supplementary.pdf]

Supplementary Materials

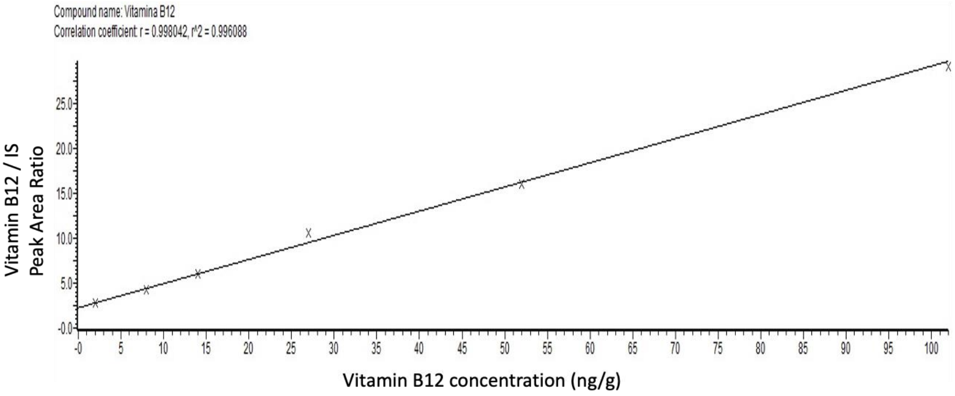

Figure S1. Matrix-matched calibration curve of Vitamin B12 in ripened cheese

Table S1. Concentration of vit. B12 measured in each ripened cheese sample

Cheese samples		Concentrations in analysis batches (ng/g)		
**Type**	**Original name**	**n.1**	**n.2**	**n.3**
Cow cheese	Caciocavallo	6.5	5.4	5.7
	Provolone	13.7	15.4	13.4
	Piave	5.6	4.6	6.2
	Grana	4.4	5.3	4.8
	Tête de moine	2.2	<LOQ	2.7
Sheep cheese	Pecorino sardo 1	17.9	21.1	24.0
	Pecorino sardo 2	20.1	28.6	18.8
	Pecorino toscano 1	40.0	40.6	36.1
	Pecorino toscano 2	37.6	30.0	25.6
Goat cheese	Caprino 1	<LOQ	<LOQ	<LOQ
	Caprino 2	<LOQ	<LOQ	<LOQ
	Caprino 3 (extra ripened)	30.6	34.7	35.3
